# Supplementary material for: Cloud BioLinux: pre-configured and on-demand bioinformatics computing for the genomics community
Source: BMC Bioinformatics. 2012 Mar 19;13:42. doi: 10.1186/1471-2105-13-42 (PMC3372431; doi:10.1186/1471-2105-13-42)
Supplement: Additional file 1 — Supplementary 1 Cloud BioLinux software documentation in the form of a mini, self-contained website. Users need to download and uncompress the .zip file, and open through a web browser the "index.html" file available on the main directory. (ZIP 1823 kb). [file 1471-2105-13-42-S1.ZIP › Cloud-BioLinux-Package-Documentation/docs/mcmctree.html]

Bio-Linux Software Documentation Pages

Back to search form

## mcmctree

|  |  |
| --- | --- |
| Name | mcmctree |
| Description | **mcmctree** is a part of the PAML package, which is a suite of programs for phylogenetic analyses of DNA or protein sequences using maximum likelihood (ML).  **References:**  Adachi, J., and M. Hasegawa. 1996a. MOLPHY Version 2.3: Programs for molecular phylogenetics based o­n maximum likelihood. Computer science monographs, 28:1-150. Institute of Statistical Mathematics, Tokyo.    Adachi, J., and M. Hasegawa. 1996b. Model of amino acid substitution in proteins encoded by mitochondrial DNA. Journal of Molecular Evolution 42:459-468. |
| Homepage | http://abacus.gene.ucl.ac.uk |
| Remote Documentation | http://abacus.gene.ucl.ac.uk/software/pamlDOC.pdf |
